# Advances in research on the protective mechanisms of traditional Chinese medicine (TCM) in myocardial ischaemia-reperfusion injury

**DOI:** 10.1080/13880209.2022.2063342

**Published:** 2022-05-19

**Authors:** Jiexin Zhang, Yonghe Hu, Han Wang, Jun Hou, Wenjing Xiao, Xudong Wen, Tingting Wang, Pan Long, Hezhong Jiang, Zhanhao Wang, Huawei Liu, Xin Chen

**Affiliations:** a Department of Laboratory Medicine, The Third People’s Hospital of Chengdu/Affiliated Hospital of Southwest, Jiaotong University, Chengdu, Sichuan, China; b Department of Central Laboratory, The General Hospital of Western Theater Command, Chengdu, Sichuan, China; c Department of Gastroenterology, The First People’s Hospital of Chengdu, Chengdu, Sichuan, China; d Faculty of Life Sciences and Engineering, Southwest Jiaotong University, Chengdu, Sichuan, China

**Keywords:** Compositae, flavonoids, antioxidative, anti-inflammatory, Chinese compound prescriptions

## Abstract

**Context:**

Developing effective drugs to treat myocardial ischaemia-reperfusion (MI/R) injury is imperative. Traditional Chinese medicines (TCMs) have had considerable success in the treatment of cardiovascular diseases. Elucidating the mechanisms by which TCMs improve MI/R injury can supplement the literature on MI/R prevention and treatment.

**Objective:**

To summarise TCMs and their main protective mechanisms against MI/R injury reported over the past 40 years.

**Methods:**

Relevant literature published between 1980 and 2020 in Chinese and English was retrieved from the Web of Science, PubMed, SpringerLink, PubMed Central, Scopus, and Chinese National Knowledge Infrastructure (CNKI) databases. Search terms included ‘medicinal plants’, ‘myocardial ischaemia reperfusion injury’, ‘Chinese medicine prescriptions’, ‘mechanisms’, ‘prevention’, ‘treatment’ and ‘protection’. For inclusion in the analysis, medicinal plants had to be searchable in the China Medical Information Platform and Plant Database.

**Results:**

We found 71 medicinal species (from 40 families) that have been used to prevent MI/R injury, of which Compositae species (8 species) and Leguminosae species (7 species) made up the majority. Most of the effects associated with these plants are described as antioxidant and anti-inflammatory. Furthermore, we summarised 18 kinds of Chinese compound prescriptions, including the compound Danshen tablet and Baoxin pill, which mainly reduce oxidative stress and regulate mitochondrial energy metabolism.

**Discussion and conclusions:**

We summarised TCMs that protect against MI/R injury and their pharmacological mechanisms. This in-depth explanation of the roles of TCMs in MI/R injury protection provides a theoretical basis for the research and development of TCM-based treatment drugs.

## Introduction

Myocardial ischaemia-reperfusion (MI/R) injury refers to the progressive aggravation of damaged tissue after blood flow to the ischaemic myocardium, which may eventually lead to myocardial fibrosis, heart failure, and myocardial infarction (Neri et al. 2017; Bai et al. 2019). Jennings et al. (1960) first identified MI/R injury. Since then, MI/R injury mechanisms and treatment strategies have been popular research topics. Currently, reperfusion injury is considered the major complication of vascular reperfusion therapy for acute myocardial infarction (MI) and is estimated to occur in up to 60% of patients (Moon et al. 2020). Inhibition of reperfusion injury is key to the treatment of MI.

The pathophysiological changes caused by reperfusion include inflammation, oxidative stress, intracellular Ca^2+^ overload, and impaired energy metabolism (Yellon and Hausenloy 2007) and ultimately can cause irreversible cell death (Heusch et al. 2010). The interrelationships of injury mechanisms often trigger or indirectly aggravate other injury factors (Garcia-Dorado et al. 2009; Yang et al. 2018). When reperfusion lasts for a few minutes, a large amount of oxygen suddenly enters the reperfused myocardium, and multiple mechanisms, such as neutrophil respiration burst and mitochondrial electron transport chain damage, lead to a sudden increase in reactive oxygen species (ROS) (Goldhaber and Weiss 1992). ROS can inhibit mitochondrial oxidative phosphorylation, resulting in insufficient energy synthesis (Laskey 2005) and mediating sarcoplasmic reticulum dysfunction (Hausenloy and Yellon 2013). Oxygen-free radicals also promote the formation of microthrombi (Ma et al. 2006). Another important mechanism is the inflammatory response, which accompanies the entire process (Marchant et al. 2012) and is the basis of myocardial structural and functional defects. Inflammation is related to the generation of ROS. Proteases and danger-associated molecular patterns (DAMPs) are released when ROS levels surge (Marchant et al. 2012), promoting inflammation by activating NF-κB (Vallabhapurapu and Karin 2009). ROS can also activate NLRP3 (Pellegrini et al. 2019) and further promote the production of inflammatory cytokines and other molecules such as IL-1β (Marchant et al. 2012), IL-6 (Legendre et al. 2005), IL-8 (Pawlinski et al. 2007), TNF-α (Saito et al. 2012), NO (Su et al. 2016) and HMGB1 (Xu et al. 2011; Herzog et al. 2014). In the acute ischaemic phase, the increase in intracellular Ca^2+^ in cardiomyocytes may be related to Ca^2+^ uptake disorders caused by Na^+^- Ca^2+^ exchange and sarcoplasmic reticulum injury (Ma et al. 2006). Furthermore, large amounts of Ca^2+^ are deposited in the mitochondria (Hausenloy and Yellon 2013), which destroys excitation-contraction coupling (Xie and Weiss 2009) and mitochondrial function, producing energy barriers. During reperfusion, Ca^2+^ and ROS activate the mitochondrial permeability transition pore (MPTP), a non-selective channel in the inner mitochondrial membrane that plays a key role in MI/R injury (Cheng et al. 2016); this activation prompts the MPTP to open, dephosphorylate (Kulek et al. 2020) and depolarise the mitochondrial membrane potential (Cheng et al. 2016), which further hinders the synthesis of ATP, causing a vicious cycle.

Nonetheless, the mechanisms of MI/R injury remain unclear, and the development of therapeutic approaches for reperfusion injury has been disappointing (Ibáñez et al. 2015). Many clinical trials have failed to demonstrate the existence of specific therapies that can reduce reperfusion injury (Fernández-Jiménez and Ibanez 2015; Jones et al. 2015). Although currently used drugs such as statins (Mensah et al. 2005) and ACE inhibitors (ACEIs) (Manning and Vehaskari 2005) have certain therapeutic effects on MI/R injury, synthetic drugs can cause side effects. The field of traditional Chinese medicine (TCM) has a history of more than 2000 years and features unique theories and abundant resources (Hao et al. 2015). Over the past several years, more than 100 TCM studies have been registered with ClinicalTrials.gov. Evidence from randomised controlled trials (Hao et al. 2017) and some other studies have indicated that TCMs can effectively relieve abnormal myocardial perfusion by acting on multiple pathways (Li et al. 2016) and controlling risk factors for cardiovascular disease. Moreover, the side effects of medicinal plants are usually mild (Sedighi et al. 2019). Therefore, it is feasible to treat MI/R injury with TCMs and their active compounds.

This review summarises both single Chinese herbs and TCM compound prescriptions that have therapeutic and protective effects against MI/R injury with a focus on the protective mechanisms. Potential medicinal plants with similar pharmacological effects are also summarised. Further research on the treatment of MI/R injury and the mechanisms of the effects of TCMs on MI/R injury is warranted.

## Methods

To carry out this review, articles in Chinese and English from 1980 to 2020 related to the treatment of MI/R injury with TCMs were retrieved from the Web of Science (WOS), PubMed, SpringerLink, PubMed Central, Scopus, and China National Knowledge Infrastructure (CNKI) databases. The search terms included ‘medicinal plants’, ‘myocardial ischaemia reperfusion injury’, ‘Chinese medicine compounds’, ‘mechanisms’, ‘prevention’, ‘treatment’, and ‘protection’. A total of 5285 articles were located. Other medicinal plants (such as Indian herbs) and Chinese medicines that have been studied repetitively were excluded. Only medicinal plants that could be retrieved from the China Medical Information Platform and Plant Database were included in the analysis. After screening, we identified articles that contained information on 18 TCM compound prescriptions and 71 species (from 40 families) of single Chinese herbs, including two plants with potential therapeutic effects.

## Results and discussion

**Figure 1.**
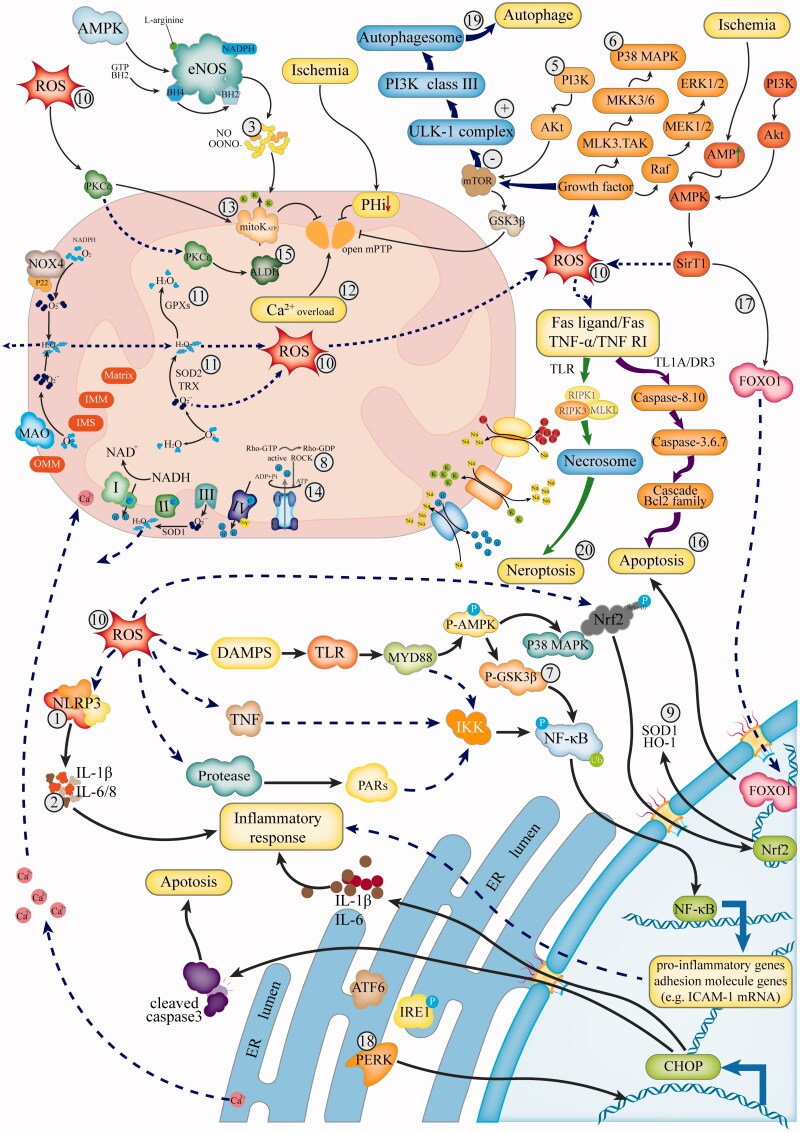
Overview of the pathophysiological mechanisms in MI/R injury affected by TCMs. ① Inhibit the NLRP3 inflammasome: *Carthamus tinctorius* L., *Erigeron breviscapus* (Vant.) Hand.-Mazz., *Artemisia annua* L. ② Reduce production of inflammatory cytokines, such as TNF-α, IL-6, IL-8: *Eclipta prostrata* L., *Bidens Pilosa* L., *Pueraria lobata* (Willd.) Ohwi, *Glycyrrhiza uralensis* Fisch., *Plumbago zeylanica* L., *Rosa rugosa* Thunb., *Dioscorea zingiberensis* C. H. Wright, *Valeriana officinalis* L., *Tribulus terrestris* L. ③ Regulate the release of NO: *Eclipta prostrata* L., *Rubiayunnanensis* (Franch.) Diels, *Fagopyrum tataricum* (L.), *Rhododendron simsii* Planch. ④ Block NF-κB activation pathway: *Erigeron multiradiatus* (Lindl.) Benth., *Bacopa monnieri* (Linn.) Wettst., *Gynostemma pentaphyllum* (Thunb.) Makino, *Cyperus rotundus* L., *Sinomenium acutum* (Thunb.) Rehd. et Wils., *Astragalus membranaceus* (Fish.) Bge., *Abelmoschus manihot* L. ⑤ Through PI3K/Akt signalling pathways: *Salvia miltiorrhiza* Bge., *Fibraurea recisa* Pierre., *Citrus reticulata* blanco, *Cyperus rotundus* L., *Myrica rubra* (Lour.) Sieb.et Zucc., *Ginkgo biloba* L., *Ilex pubescens* Hook.et Arn. ⑥ Block p38/ERK MAPK pathway: *Bacopa monnieri* (Linn.) Wettst., *Fibraurea recisa* Pierre., *Gynostemma pentaphyllum* (Thunb.) Makino, *Cyperus rotundus* L., *Draba nemorosa* L., *Gastrodia elata* Bl., *Diospyros kaki* Thunb. ⑦ Activate AMPK/GSK-3β signalling pathway: *Carthamus tinctorius* L., *Aloe barbadensis* Miller. ⑧ Regulate RhoA signalling pathway: *Panax ginseng* C. A. Mey. ⑨ Activate the Nrf2/ARE/HO-1 signal pathway: *Dalbergia odorifera* T. Chen., *Rheum palmatum* L. ⑩ Scavenging ROS: *Cassia mimosoides* L., *Bidens pilosa* L., *Arctium lappa* L., *Fagopyrum tataricum* (L.), *Panax notoginseng* (Burk.) F. H. Chen, *Plumbago zeylanica* L., *Magnolia officinalis* Rehd.et Wils., *Acanthopanax senticosus* (Rupr.et maxim) Harms, *Lentinus edodes* (Berk.) Sing., *Cuscuta chinensis* Lam., *Bombax malabaricum* L., *Tribulus terrestris* L., *Rhododendron simsii* Planch. ⑪ Enhance the activity of antioxidant enzymes, such as SOD, CAT, glutathione peroxidase: *Diospyros kaki* Thunb., *Glycyrrhiza uralensis* Fisch., *Sophora japonica* L., *Bauhinia championii* (Benth.) Benth., *Eclipta prostrata* L., *Silybum marianum* (Linn.) Gaertn., *Polygonum multiflorum* Thunb., *Citrus maxima* (Burm.) Merr., *Panax notoginseng* (Burk.) F. H. Chen, *Rosa rugosa* Thunb., *Schisandra chinensis* (Turcz.) Ball, *Galium verum* L., *Andrographis paniculata* (Burm.F.) nees, *Valeriana officinalis* L., *Cinnamomum cassia* Presl, *Malva sinensis* Cav. ⑫ Reduce Ca^2+^ overload: *Mollugo pentaphylla* L. ⑬ Activate the mitoKATP channel: *Citrus maxima* (Burm.) Merr., *Dioscorea zingiberensis* C. H. Wright, *Hypericum monogynum* L. ⑭ Improve mitochondrial energy metabolism: *Crataegus pinnatifida* Bge., *Dracocephalum moldavica* L., *Polygonatum odoratum* (Mill.) Druce, *Rubiayunnanensis* (Franch.) Diels. ⑮ Activate ALDH2 to protect mitochondrial function: *Piper longum* Linn. ⑯ Prevent the apoptotic cascade: *Cassia mimosoides* L., *Astragalus membranaceus* (Fish.) Bge., *Pueraria lobata* (Willd.) Ohwi, *Curcuma longa* L., *Tribulus terrestris* L., *Morus alba* L. ⑰ Activate sirtuin-1/FOXO1 signal: *Epimedium brevicornu* Maxim., *Paeonia veitchii* Lynch. ⑱ Inhibit ATF6 and PERK pathways to attenuate ERS: *Dracaena cochinchinensis* (Lour.) S. C. Chen, *Allium fistulosum* L., *Schisandra chinensis* (Turcz.) Ball, *Paeonia lactiflora* Pall. ⑲ Inhibit autophagy: *Coptis chinensis* Franch., *Magnolia officinalis* Rehd.et Wils., *Gardenia jasminoides* Ellis. ⑳ Inhibit necrosis: *Scutellaria baicalensis* Ceorgi, *Arctium lappa* L., *Bauhinia championii* (Benth.) Benth.

The mechanism of MI/R injury is complicated and involves inflammation, oxidative stress, intracellular Ca^2+^ overload, impairment of energy metabolism and ultimately irreversible cell death, as described previously (Yellon and Hausenloy 2007; Heusch et al. 2010). We identified 71 species (from 40 families) of single Chinese herbs used in MI/R treatment and classified them according to their pharmacological mechanisms (Tables 1–5). Two potential plants with similar pathological effects (Table 6) and 18 types of TCM compound prescriptions (Table 7) were also summarised. An overview of the possible mechanisms underlying the effects of TCMs in the treatment of MI/R injury is shown in Figure 1. Next, we will review these TCMs in terms of their protective mechanisms against MI/R injury.

**Table 1.** Chinese herbs that are considered to have anti-inflammatory effects in MI/R injury.

Family	Latin binomial	Part used	Active compounds	Dose	Experimental animal model	Pharmacological mechanisms	References
Araliaceae	*Panax ginseng* C. A. Mey.	Roots	Ginsenoside Rb1 (Rb1)	2.5, 5, 7.5 mg/kg	Male SD rats, 30 min of ischaemia and 90 min of reperfusion.	Regulate RhoA signalling pathway.	Cui et al. 2017
Brassicaceae	*Draba nemorosa* L.	Seeds	Aqueous extract	5 × 10^3^, 10 × 10^3^, 20 × 10^3^ mg/kg	Male SD rats, 30 min of ischaemia and 2 h of reperfusion.	Through MAPK/ERK1/2 pathway.	Chen et al. 2019
Compositae	*Erigeron multiradiatus* (Lindl.) Benth.	Whole	Caffeoylquinic Acid Derivatives Extract (AE)	10, 20, 40 mg/kg	Male SD rats, 30 min of ischaemia and 24 h of reperfusion.	Block NF-κB and JNK activation pathway	Zhang et al. 2016
	*Carthamus tinctorius* L.	Flowers	Hydroxysafflor yellow A (HSYA)	4, 8, 16 mg/kg	Male SD rats, 30 min of ischaemia and 24 h of reperfusion.	Inhibit the NLRP3 inflammasome.	Ye et al. 2020
				6.25, 12.5, 25 µM	H9c2 cardiomyocytes hypoxia for 6 h then reoxygenation.		
	*Erigeron breviscapus* (Vant.) Hand.-Mazz.	Flowers	Scutellarin (Scu)	5, 10, 20 mg/kg	Male SD rats, 30 min of ischaemia and 24 h of reperfusion	Regulating the Akt/mTORC1/NLRP3 signalling pathway.	Xu et al. 2020
				3.125, 6.25, 12.5 µg/ml	H9c2 cardiomyocytes hypoxia for 4 h then reoxygenation 24 h.		
	*Artemisia annua* L.	Dry aerial part	Artemisinin (ARS)	7 mg/kg	Male SD rats, 30 min of ischaemia and 2 h of reperfusion.	Inhibit the activation of NLRP3 inflammasome.	Wang et al. 2020)
	*Eclipta prostrata* L.	Dry aerial part	Aqueous extract	0.5 × 10^3^, 10 × 10^3^ mg/kg	Male SD rats, 30 min of ischaemia and 2 h of reperfusion	Regulate the release of TNF-α, IL-6, NO	Jia 2014
	*Bidens pilosa* L.	Dry aerial part	Bidens flavonoids (TFB)	40, 80, 160 mg/kg	Male Wistar rats, 30 min of ischaemia and 2 h of reperfusion	Reduce the production of TNF-α, IL-8.	Ma et al. 2015
Cucurbitaceae	*Gynostemma pentaphyllum* (Thunb.) Makino	Whole grass	Gypenoside (GP)	50, 100, 200 mg/kg	Male Wistar rats, ischaemia for 45 min plus 3 h reperfusion.	Inhibit NF-κB p65 activation through MAPK signalling pathway.	Yu et al. 2016
				5, 10, 20 µM	Oxygen-glucose deprivation–reoxygenation (OGD/R) H9c2 cell model.		
Cyperaceae	*Cyperus rotundus* L.	Rhizomes	Nootkatone	10 mg/kg	Male Wistar albino rats by subcutaneous injection of ISO (85 mg/kg).	Mitigating inflammation by modulating altered TLR4/NF-κB/MAPK signalling.	(Meeran et al. 2021)
Dioscoreaceae	*Dioscorea zingiberensis* C. H. Wright	Rhizomes	Diosgenin	0.001 μM	Male Wistar rats, ischaemia for 30 min and reperfused for 90 min with langdorff.	Reduce the production of inflammatory mediators.	(Ebrahimi et al. 2014)
Ericaceae	*Rhododendron simsii* Planch.	Flowers	Total Flavonoids (TFR)	10, 20, 40 mg/kg	In SD male rats, 30 min of ischaemia and 60 min of reperfusion.	Increase the production of NO.	(Zhang JH and Chen 2007)
Labiatae	*Salvia miltiorrhiza* Bge.	Dry roots	Salvianolic acid B (Sal B)	15, 60 mg/kg	Male SD rats, 30 min of ischaemia and 24 h of reperfusion.	Inhibit the expression of HMGB1 of the PI3K/Akt signalling pathway.	(Liu et al. 2020)
Leguminosae	*Astragalus membranaceus* (Fish.) Bge	Dry roots	Astragaloside IV (AsIV)	20, 40, 80 mg/kg	Male SD rats, 30 min of ischaemia and 120 min of reperfusion.	Inhibit TLR4/NF-κB signalling pathway and reduce serum inflammatory factors.	(Lu et al. 2015)
	*Glycyrrhiza uralensis* Fisch.	Dried roots and rhizomes	Isoliquiritin	25, 50, 75 mg/mL	Establish the isolated cardiac perfusion model by langendorff.	Regulate the release of TNF-α, IL-6 and CPR.	(Ren et al. 2016)
	*Pueraria lobata* (Willd.) Ohwi	Flowers	Total Flavonoids	20, 40, 60 mg/kg	SPF male Wistar rats, 30 min for reperfusion after ischaemia.	Reduce infiltration of inflammatory cytokines.	(Fan HX and Zhang et al. 2017)
Liliaceae	*Aloe barbadensis* Miller	Leaf juice	Barbaloin (BAR)	20 mg/kg	Male SD rats, 30 min of ischaemia and 3 h of reperfusion.	Balance inflammation response through AMPK activation.	(Zhang et al. 2017)
Malvaceae	*Abelmoschus manihot* (Linn.) Medicus	Dried corolla	Total flavone (TFA)	4, 8, 16 mg/kg	Rabbits, 30 min of ischaemia and 60 min of reperfusion.	Inhibit the high expression of ICAM-1mRNA.	(Fan et al. 2006)
Menispermaceae	*Cocculus trilopus* (Thunb.) DC.	Cane	Sinomenine	15, 30, 60 mg/kg	SD rats, 30 min of ischaemia and 2 h of reperfusion.	Inhibit the release of inflammatory factors and inhibit the TLR4 /NF-κBp65 pathway.	(Xu F 2018)
Plumbaginaceae	*Plumbago zeylanica* L.	Dry roots	Plumbagin	5 mg/kg	Male C57BL6/J mice, 45 min of ischaemia and 4 h of reperfusion.	Induce Nrf2 activation and reduce cytokine expression.	(Wang et al. 2016)
Polygonaceae	*Fagopyrum tataricum* (L.)	Roots	Flavonoids	50mg/kg	SD rats, 45 min of ischaemia and 60 min of reperfusion.	Increase the level of NO.	(Pan et al. 2015)
Rosaceae	*Rosa rugosa* Thunb.	Dry flower buds	Xinjiang sprig rose total flavonoid (XSRTF)	5, 10, 20 g/mL	SD rats, ischaemia for 20 min and reperfused for 45 min with langendorff device.	Reduce the production of CRP, IL-8, IL-6, and TNF-α,	(Hou et al. 2016)
Rubiaceae	*Rubiayunnanensis* (Franch.) Diels	Dried roots and rhizomes	Ethanolic extract	56.7, 170, 280 mg/kg	Male Wistar rats, 30 min of ischaemia and 2 h of reperfusion.	Increase serum NO level.	(Zhang et al. 2019)
Scrophulariaceae	*Bacopa monnieri* (Linn.) Wettst.	Whole grass	Ethanol extract	30, 100 μg/ ml	Establish the isolated cardiac perfusion model by langendorff.	Block the inflammatory transcription factor NF-kB or p38/ERK MAPK pathway.	(Srimachai et al. 2017)
Tetrandrae	*Fibraurea recisa* Pierre.	Dry rattan	Fibrauretine	50, 100, 200 mg/kg	Male SD rats, 30 min of ischaemia and 2 h of reperfusion.	Through the PI3K/Akt and ERK 1/2 signal pathways.	(Wang et al. 2020)
Valerianaceae	*Valeriana officinalis* L.	Roots and rhizomes	Valerian extract	100 mg/kg	Big-eared white rabbits, 1 h of ischaemia and 1.5 h of reperfusion.	Reduce the production of TNF-α.	(Yin et al. 2000)
Zygophyllaceae	*Tribulus terrestris* L.	Whole grass	Gross saponins of Tribulus terrestris (GSTT)	10, 30, 100 mg/kg	Ischaemia for 30 min and reperfusion for 2 h.	Reduce the production of inflammatory factors.	(Zhang et al. 2010)

**Table 2.** Chinese herbs that are considered to inhibit oxidative stress in MI/R injury.

Family	Latin Binomial	Part Used	Active Compounds	Dose	Experimental animal model	Pharmacological mechanisms	References
Acanthaceae	*Andrographis paniculata* (Burm.F.) nees	Dried aerial part	Hydroalcoholic extract	200 mg/kg	Male Wistar albino rats, 45 min of ischaemia and 1 h of reperfusion.	Increase the activity of SOD and CAT.	(Ojha et al. 2012)
Apiaceae	*Panax notoginseng* (Burk.) F. H. Chen	Dry roots	Notoginsenoside R1（NGR1）	5, 10, 20 μ M	Male SD rats, 40 min of ischaemia and 60 min of reperfusion with langendorff.	Inhibit oxidative stress and ERS related apoptosis.	(Yu et al. 2016)
Araliaceae	*Acanthopanax senticosus* (Rupr.et maxim) Harms	Dried roots and rhizomes	Acanthopanax senticosus saponins (ASS)	25, 50, 100 mg/kg	Ischaemia for 30 min and reperfusion for 2 h.	Reduce the level of free radicals, improve myocardial metabolism.	(Sui et al. 2004)
Berberidaceae	*Epimedium brevicornu* Maxim.	Leaves	Icariin	60 mg/kg	Ischaemia for 30 min and reperfusion for 24 h.	Activate sirtuin-1 / FOXO1 signal and reduce oxidative stress.	(Wu et al. 2018)
Bombacaceae	*Bombax malabaricum* L.	Flowers	Total flavonoids (TFG)	100, 200, 400 mg/kg	Male SD rats, 30 min of ischaemia and 60 min of reperfusion.	Reduce the generation of oxygen free radicals and adjust the balance of oxidation and anti-oxidation.	(Lu et al. 2020)
Compositae	*Arctium lappa* L.	Dried ripe fruits	Arctiin	15, 30, 60 mg/kg	Male SD rats were suffered ischaemia for 1 h plus 3 h-reperfusion.	Remove active oxygen.	(Chen et al. 2020)
				10, 20, 40 μM	The H9c2 rat cardiomyocyte cell line was hypoxia for10 h and reoxygenation for 4 h.		
	*Bidens pilosa* L.	Dry aerial part	Bidens flavonoids (TFB)	40, 80, 160 mg/kg	Male Wistar rats received ischaemia for 30 min and reperfused for 2 h.	Has anti-free radical effects.	(Ma et al. 2015)
	*Eclipta prostrata* L.	Dry aerial part	Aqueous extract	0.5 × 10^3^, 10 × 10^3^ mg/kg	Male SD rats were ischaemic for 30 min and reperfused for 2 h.	Improve SOD activity.	(Jia D 2014)
	*Silybum marianum* (Linn.) Gaertn.	Dried ripe fruits	Silybin (SIL)	100, 200, 400 mg/kg	Male SD rats, 30 min of ischaemia and 6 h of reperfusion.	Significantly increase the activity of SOD and CAT.	(Cao et al. 2017)
Convolvulaceae	*Cuscuta chinensis* Lam.	Dried mature seeds	Ethanolic extract	12.5 g/L	For the isolated heart of male SD rats, first cause 20 min of ischaemia, then reperfusion with K-H solution for 45 min.	Improve the ability of cardiomyocytes to scavenge free radicals.	(Yu et al. 2013)
Cyperaceae	*Cyperus rotundus* L.	Rhizomes	Nootkatone	10 mg/kg	Male Wistar albino rats by subcutaneous injection of ISO (85 mg/kg).	Mitigating oxidative stress, by activating PI3K/Nrf2/Akt signalling cascades.	(Meeran et al. 2021)
Ebenaceae	*Diospyros kaki* Thunb.	Leaves	Persimmon leaf extract (PEL)	50 mg/kg	Male SD rats, 30 min of ischaemia and 60 min of reperfusion.	Increase the vitality of SOD and GSH-Px.	(Meng et al. 2019)
Ericaceae	*Rhododendron simsii* Planch.	Whole grass	Total Flavonoids (TFR)	10, 20, 40 mg/kg	In SD male rats, 30 min of ischaemia and 60 min of reperfusion.	Reduce the peroxidation of free radicals.	(Zhang JH and Chen 2007)
Lauraceae	*Cinnamomum cassia* Presl	Dried bark	Ethanolic extract	50, 100, 200 mg/kg	Male SD rats, ischaemia for 30 min and reperfusion for 5 days.	Increase the activity of SOD and CAT, decrease MDA.	(Sedighi et al. 2018)
Leguminosae	*Bauhinia championii* (Benth.) Benth.	Rhizomes	Flavones (BCF)	20 mg/kg	For SD rats, 30 min of ischaemia, 12 h and 24 h of reperfusion.	Improve total antioxidant capacity (T-AOC).	(Zhang et al. 2016)
	*Cassia mimosoides* L.	Whole plants	Methanol Extract	400 mg/kg	For male SD rats, 30 min of ischaemia and 3 h of reperfusion.	Scavenging ROS.	(Lim and Lee 2012)
	*Dalbergia odorifera* T. Chen	Dry heartwood of trunk and roots	latifolin	2. 5, 5, 10, 20 μg /mL	The H9c2 rat cardiomyocyte cell was hypoxia for 2 h and reoxygenation for 3 h.	Activating Nrf2 /HO-1 signalling pathway to reduce the level of ROS.	(Zhang et al. 2019)
	*Glycyrrhiza uralensis* Fisch.	Dried roots and rhizomes	Isoliquiritin	25, 50, 75 mg/mL	N/A	Significantly increase the activity of SOD and the ratio of GSH/GSSG, and reduce the content of MDA.	(Ren et al. 2016)
	*Sophora japonica* L.	Dry flowers and buds	Rutin	50 µM	The isolated hearts of male SD rats were subjected to global ischaemia for 20 min and reperfusion for 50 min.	Enhancing the activity of SOD and DPPH.	(Bhandary et al. 2012)
				1, 10, 100 µM	N/A		
Liliaceae	*Aloe barbadensis* Miller	Leaf juice	Barbaloin (BAR)	20 mg/kg	Male SD rats, 30 min of ischaemia and 3 h of reperfusion.	Balance oxidative stress through AMPK activation.	(Zhang et al. 2017)
Magnoliaceae	*Magnolia officinalis* Rehd.et Wils.	Bark	Honokiol (HKL)	5, 10, 20, 40, or 80 μM	In vitro H/R model, 3 h hypoxia and 3 h reoxygenation.	Reduce intracellular ROS production.	(Tan et al. 2019)
	*Schisandra chinensis* (Turcz.) Ball	Fruits	Schisandrin B (Sch B)	20, 40, 80 mg/kg	Male SD rats, 40 min of ischaemia and 1 h of reperfusion.	Improve SOD activity and decrease MDA.	(Zhang et al. 2017)
Malvaceae	*Malva sinensis* Cav.	Flowers	Methanol extract	250, 500 mg/kg	Male SD rats were ischaemic for 30 min and reperfused for 2 h.	Increase the level of SOD and CAT.	(Zuo 2017)
Myricaceae	*Myrica rubra* (Lour.) Sieb.etZucc.	Bark	Myrica rubra flavonoids (MRF)	5, 10, 20mg/kg	SD rats injected with ISO (4 mg/kg).	Regulate PI3K/Akt/GSK3β pathway.	(Wang et al. 2019)
				6.25 µg/ml	H9c2 cardiomyocyte cell was hypoxia for 6 h and reoxygenation for 12 h.		
Plumbaginaceae	*Plumbago zeylanica* L.	Dry roots	Plumbagin	5 mg/kg	Male C57BL6/J mice, 45 min of ischaemia and 4 h of reperfusion.	Reduce oxidative stress by reducing ROS and lipid peroxide levels.	(Wang et al. 2016)
Polygonaceae	*Fagopyrum tataricum* (L.)	Roots	Flavonoids	50 mg/kg	Clean SD rats, 45 min of ischaemia and 60 min of reperfusion.	Improve the scavenging ability of oxygen free radicals, inhibit the generation of oxygen free radicals.	(Pan et al. 2015)
	*Polygonum multiflorum* Thunb.	Dried roots	Ethyl acetate extract (PME)	10^3^, 2 × 10^3^ mg/kg	Female SD rats, ischaemia for 10 min and reperfused for 15 min with langendorff.	Maintain the antioxidant status of glutathione.	(Yim et al. 1998)
	*Rheum palmatum* L.	Rhizomes	Emodin	20, 40, 60 mg/kg	Male SD rats were reperfused for 2 h after 30 min of ischaemia.	Activate the Nrf2 / ARE / HO-1 signal pathway.	(Cui et al. 2020)
Rosaceae	*Rosa rugosa* Thunb.	Dry flower buds	Xinjiang sprig rose total flavonoid (XSRTF)	5, 10, 20 g/mL	SD rats, ischaemia for 20 min and reperfused for 45 min with langendorff device.	Decrease in SOD activity and reduce ROS concentration.	(Hou et al. 2016)
Rubiaceae	*Galium verum* L.	Whole grass	Methanol extract	500 mg/kg	Male spontaneously hypertensive rats, ischaemia for 20 min and reperfused for 30 min.	Enhance the activity of myocardial SOD and reduce the production of cardiac O^2-^.	(Bradic et al. 2019)
Rutaceae	*Citrus reticulata* Blanco	Ripe peel	Nobiletin	15 mg/kg	Male C57BL/6 mice were reperfused for 2 h after 30 min of ischaemia.	Activate the PI3K-Akt pathway and reduce oxidative stress.	(Chen et al. 2016)
	*Citrus maxima* (Burm.) Merr.	Dry outer layer of grapefruit	Naringenin (Nari)	1.25, 2.5, 5, 10, 20, or 40 µmol/L	Male SD rats, 30 min of ischaemia, then reperfused for 60 min with langendorff.	Improve SOD activity and decrease MDA.	(Meng et al. 2016)
Tricholomataceae	*Lentinus edodes* (Berk.) Sing.	Fruits	Lentinan (LNT)	15, 30, 60 mg/kg	Male SD rats, 60 min of ischaemia and 30 min of reperfusion.	Anti-free radicals, protect the activity of oxygen free radical scavenging enzymes, and reduce lipid peroxidation.	(Chen J 2014)
Valerianaceae	*Valeriana officinalis* L.	Roots and rhizomes	Valerian extract	100 mg/kg	For big-eared white rabbits, 1 h of ischaemia and 1.5 h of reperfusion.	Inhibit xanthine oxidase, reduce the production of free radicals, increase the ratio of PGI/TXA.	(Yin et al. 2000)
Zygophyllaceae	*Tribulus terrestris* L.	Flowers, leaves and above ground	Gross saponins of Tribulus terrestris (GSTT)	10, 30, 100 mg/kg	Ischaemia for 30 min and reperfusion for 2 h.	Reduce the production of free radicals.	(Zhang et al. 2010)

**Table 3.** Chinese herbs that are considered to reduce Ca^2+^ overload and regulate mitochondrial energy metabolism in MI/R injury.

Family	Latin binomial	Part used	Active compounds	Dose	Experimental animal model	Pharmacological mechanisms	References
Aizoaceae	*Mollugo pentaphylla* L.	Whole grass	Corngrass extract	6 mg/kg	Male SD rats, ischaemia for 30 min and reperfusion for 60 min.	Reduce Ca^2+^ overload.	(Li et al. 2016)
Dioscoreaceae	*Dioscorea zingiberensis* C. H. Wright	Rhizomes	Diosgenin	0.001 μM	Male Wistar rats, ischaemia for 30 min, then reperfusion for 90 min with langendorff.	Activate the mitoKATP channel.	(Ebrahimi et al. 2014)
Guttiferae	*Hypericum monogynum* L.	Roots	Hyperoside	50 mg/kg	Male SD rats, ischaemia for 30 min and reperfused for 2 h.	Activate the PKCα signalling pathway, or activate PKCε to open the mitoKATP channel.	(Wang SF 2020)
Labiatae	*Dracocephalum moldavica* L.	Whole grass	Tilianin	1.25, 2.5, 5 mg/kg	M Male SD rats, ischaemia for 30 min and reperfusion for 24 h.	Regulate myocardial energy metabolism.	(Jiang et al. 2014)
Liliaceae	*Polygonatum odoratum* (Mill.) Druce	Dried roots and rhizomes	Ethanol extract (PORE)	100, 200, 300 mg/kg	Male Wistar rats, ischaemia for 30 min, reperfused for 120 min．	Protect mitochondria and enhance energy metabolism.	(Yang et al. 2018)
Piperaceae	*Piper longum* Linn.	Dry fruits	Piperlonguminine (PPLG)	1.5 mg/kg	Male Wistar rats, ischaemia for 5 min and 5 min for reperfusion.	Activate ALDH2, and protect mitochondrial function.	(Yoval-Sánchez et al. 2020)
Rosaceae	*Crataegus pinnatifida* Bge.var.major N.E.Br.	Leaves	Vitexin	1, 3, 10 μM	Male SD rats were treated with langendorff method for 30 min of ischaemia, then reperfused for 30 min.	Regulate mitochondrial dysfunction.	(Xue et al. 2020)
Rubiaceae	*Rubiayunnanensis* (Franch.) Diels	Dried roots and rhizomes	Ethanolic extract	56.7, 170, 280 mg/kg	Male Wistar rats, 30 min of ischaemia and 2 h of reperfusion.	Improve mitochondrial energy metabolism.	(Zhang et al. 2019)
Rutaceae	*Citrus maxima* (Burm.) Merr.	Dry outer layer of grapefruit	Naringenin (Nari)	1.25, 2.5, 5, 10, 20, or 40 µmol/L	Male SD rats, 30 min of ischaemia, then reperfused for 60 min with langendorff.	Activate ATP-sensitive potassium channels.	(Meng et al. 2016)

**Table 4.** Chinese herbs that are considered to inhibit apoptosis in MI/R injury.

Family	Latin binomial	Part used	Active compounds	Dose	Experimental animal model	Pharmacological mechanisms	References
Aquifoliaceae	*Ilex pubescens* Hook.et Arn.	Roots	Ilexsaponin A	10, 40 mg/kg	20 min of ischaemia and 40 min of reperfusion.	Activating PI3K/Akt pathway.	(Zhang et al. 2017)
				10, 50, 250 μg/ml	Cardiomyocytes hypoxia for 4 h and reoxygenate for 4 h.		
Araliaceae	*Panax ginseng* C. A. Mey.	Roots	Ginsenoside Rb1 (Rb1)	2.5, 5, 7.5 mg/kg	Male SD rats were subjected to 30 min of ischaemia and 90 min of reperfusion.	Regulate RhoA/Rock signalling pathway.	(Cui et al. 2017)
Compositae	*Silybum marianum* (Linn.) Gaertn.	Dried ripe fruits	Silybin (SIL)	100, 200, 400 mg/kg	Male SD rats, 30 min of ischaemia and 6 h of reperfusion.	N/A	(Cao et al. 2017)
Ebenaceae	*Diospyros kaki* Thunb.	Leaves	Persimmon leaf extract (PEL)	50 mg/kg	Male SD rats, 30 min of ischaemia and 60 min of reperfusion.	Activating the MAPK / ERK1/2 signalling pathway.	(Meeran et al. 2021)
Ginkgoaceae	*Ginkgo biloba* L.	Leaves	Ginkgolide B	0.01, 0.1, 1, 10, 100 μM	Exposing H9c2 cells to different H2O2 concentrations (200, 400, 600 and 800 µM) and harvesting at 4, 8 and 12 h.	Activating PI3K/Akt/mTOR signalling pathway.	(Liu et al. 2020)
Leguminosae	*Astragalus membranaceus* (Fish.) Bge	Dry roots	Astragaloside IV (AsIV)	20, 40, 80 mg/kg	Male SD rats, 30 min of ischaemia and 120 min of reperfusion.	Inhibit the expression of Bax and increase the expression of Bcl-2.	(Lu et al. 2015)
	*Cassia mimosoides* L.	Whole plants	Methanol Extract (Makino)	400 mg/kg	Male SD rats, 30 min of ischaemia and 3 h of reperfusion.	Prevent the apoptotic cascade.	(Lim and Lee 2012)
	*Glycyrrhiza uralensis* Fisch.	Dried roots and rhizomes	Isoliquiritin	25, 50, 75 mg/mL	N/A	N/A	(Ren et al. 2016)
	*Pueraria lobata* (Willd.) Ohwi	Flowers	Total Flavonoids	20, 40, 60 mg/kg	Male Wistar rats, the arterial clamp was released 30 min after ischaemia to restore blood perfusion.	Inhibit the expression of Bax and increase the expression of Bcl-2.	(Fan HX and Zhang et al. 2017)
Liliaceae	*Allium fistulosum* L.	Bulb near root	Monomer sulphide S1	10, 50, 100 μg/ml	H9c2 cardiomyocytes, hypoxia for 4 h, and reoxygenate for 0, 4, and 16 h.	Attenuate ERS.	(Li Y 2019)
	*Dracaena cochinchinensis* (Lour.) S. C. Chen	Tree stem	Resina draconis	0.25, 0.5, 1.0 mg/ml	Tree shrews, 30 min of ischaemia, then remove the silicone tube to achieve reperfusion.	Attenuate ERS by regulating the miR-423-3p / ERK signalling pathway.	(Yang et al. 2019)
Magnoliaceae	*Schisandra chinensis* (Turcz.) Ball	Fruits	Schisandrin B (Sch B)	20, 40, 80 mg/kg	Male SD rats, 40 min of ischaemia and 1 h of reperfusion.	Inhibit ATF6 and PERK pathways to attenuate ERS.	(Zhang et al. 2017)
Moraceae	*Morus alba* L.	Leaves	Total flavones	35, 70, 140 mg/kg	Male SD rats, 30 min of ischaemia and 60 min of reperfusion.	Down regulate the expression of Caspase-3 protein and reduce cell apoptosis.	(Wang P 2011)
Orchidaceae	*Gastrodia elata* Bl.	Dried rhizome	Gastrodin	10, 20, 40 μmol/L	Incubate the serum-free DMEM/F12 high glucose medium for 2 h, then change to DMEM/F12 high glucose medium containing 10% calf serum for 4 h.	Activate Akt / p38MAPK signalling pathway to inhibit the expression of apoptosis-related proteins.	(Zhang et al. 2019)
Ranunculaceae	*Paeonia lactiflora* Pall.	Dried roots	Total glueosides of paeony (TGP)	50, 100, 200 mg/kg	Rats were ischaemic for 30 min and reperfused for 2 h.	Attenuate ERS.	(Zheng YP and Liu et al. 2019)
	*Paeonia veitchii* Lynch	Dried roots	Oxypaeoniflorin (OPA)	10, 20, 40 mg/kg	C57BL/6 male mice was subjected to 30 min of ischaemia and 2 h of reperfusion.	Activate Sirt1/Foxo1 signalling pathway.	(Wang K and Hu 2020)
Rosaceae	*Rosa rugosa* Thunb.	Dry flower buds	Xinjiang sprig rose total flavonoid (XSRTF)	5, 10, 20 g/mL	SD rats, ischaemia for 20 min and reperfused for 45 min with using langdorff device.	N/A	(Hou et al. 2016)
Zingiberaceae	*Curcuma longa* L.	Dried rhizome	Aqueous extract	100 mg/kg	Male Wistar rats, 45 min of ischaemia and 1 h of reperfusion.	Inhibit the expression of Bax and increase the expression of Bcl-2.	(Mohanty et al. 2006)
Zygophyllaceae	*Tribulus terrestris* L.	Whole grass	Gross saponins of Tribulus terrestris (GSTT)	10, 30, 100 mg/kg	Ischaemia for 30 min and reperfusion for 2 h.	Inhibit the expression of Bax and increase the expression of Bcl-2.	(Zhang et al. 2010)

**Table 5.** Chinese herbs that are considered to inhibit necrosis and autophagy in MI/R injury.

Pharmaceutical effect	Family	Latin binomial	Part used	Active compounds	Dose	Experimental animal model	Pharmacological mechanisms	References
Inhibit necrosis.	Compositae	*Arctium lappa* L.	Dried ripe fruits	Arctiin	15, 30, 60 mg/kg	Male SD rats, ischaemia for 1 h plus 3 h reperfusion.	Inhibiting the production of necrose-related proteins.	(Chen et al. 2020)
					10, 20, 40 μM	H9c2 cardiomyocyte cell was hypoxia for10 h and reoxygenation for 4 h.		
	Labiatae	*Scutellaria baicalensis* Ceorgi	Dry roots	Baicalein	25 mg/kg	Male C57BL/6 mice was subjected to 30 min of ischaemia and 3 h of reperfusion.	Degrade the expression of necrosis-related proteins RIPK1 and RIPK3 and inhibit the formation of necrosis complexes.	(Wang et al. 2020)
	Leguminosae	*Bauhinia championii* (Benth.) Benth.	Rhizomes	Flavones (BCF)	20 mg/kg	SD rats, 30 min of ischaemia, 12 h and 24 h of reperfusion.	Down regulate the expression of RIPK 3.	(Zhang et al. 2016)
Inhibit autophagy.	Magnoliaceae	*Magnolia officinalis* Rehd.et Wils.	Bark	Honokiol (HKL)	5, 10, 20, 40, or 80 μM	*In vitro* H/R model received 3 h hypoxia and 3 h reoxygenation.	Enhance autophagy flux.	(Tan et al. 2019)
	Ranunculaceae	*Coptis chinensis* Franch.	Dried rhizome	Berberine (BBR)	50 µM	The H9c2 cells were hypoxia for 4 h and then reoxygenated for 3 h.	N/A	(Zhu et al. 2020)
	Rubiaceae	*Gardenia jasminoides* Ellis	Dried ripe fruits	Geniposide (GP)	100 mg/kg	Male SD rats, 30 min of ischaemia and 2 h of reperfusion.	Activating the AKT/mTOR signalling pathway.	(Luo et al. 2020)

**Table 6.** Potential Chinese herbs that are considered to have anti-MI/R injury properties.

Family	Latin binomial	Part used	Active compounds	Possible mechanisms against MI/R injury	References
Labiatae	*Rosmarinus officinalis* L.	Whole grass	MDX 60	Reduce the area of myocardial infarction.	(Zhang et al. 2017)
Theaceae	*Camellia oleifera* Abel	Roots	Sasanquasaponin (SQS)	Protect cardiomyocytes by regulating Cl^-^ levels in cells.	(Lai et al. 2004)

**Table 7.** TCM compounds that are considered to prevent MI/R injury.

Chinese medicine compound prescriptions	Main components	Active ingredient	Effect	References
Baoxin Pill	*Panax ginseng* C. A. Mey*., Astragalus membranaceus* (Fish.) Bge*, Ophiopogon japonicus* (Linn. f.) Ker-Gawl*., Salvia miltiorrhiza* Bge.*, Ligusticum chuanxiong* Hort.*, Acorus tatarinowii* Schott．	N/A	Reduce I/R myocardial calcium accumulation, protect mitochondrial function and inhibit the production of xanthine oxidase, prevent lipid peroxidation, etc.	(Wang et al. 1997)
Compound Danshen Tablet	*Salvia miltiorrhiza* Bge.*, Panax notoginseng* (Burk.) F. H. Chen	Tanshinone, salvianolic acid, notoginsenoside	Dilate coronary artery and enhance serum NO and eNOS levels.	(Li et al. 2020)
Compound Wenxin Decoction	*Panax ginseng* C. A. Mey*., Cinnamomum cassia* Presl*, Allium macrostemon* Bge.*, Pinellia ternata* (Thunb.) Breit.*, Trichosanthes kirilowii* Maxim.*, Paeonia veitchii* Lynch*, Ligusticum chuanxiong* Hort*, Glycyrrhiza uralensis* Fisch.	N/A	Preconditioning can significantly reduce the release of myocardial enzymes from ischaemia-reperfusion myocardium, and has the effect of resisting myocardial ischaemia and reperfusion injury arrhythmia.	(Li et al. 2004)
Dingxin Prescription	*Sophora flavescens* Ait.*, Coptis chinensis* Franch.*, Ziziphus jujuba* Mill.*, Codonopsis pilosula* (Franch.) Nannf.*, Panax notoginseng* (Burk.) F. H. Chen*, Paeonia veitchii* Lynch*, Salvia miltiorrhiza* Bge.	Matrine, Oxymatrine, Sophora flavonoids, Berberine, Jujube seed total saponins (A and B)	Block sodium and calcium channels, reduces NE, DA and 5-HT content in rat plasma and platelets, and has anti-arrhythmia caused by myocardial ischaemia and reperfusion injury.	(Jia et al. 1999)
Gualou Xiebai Decoction	*Trichosanthes kirilowii* Maxim.*, Allium macrostemon* Bge.	N/A	Removal of oxygen free radicals, inhibition of P38, JNK, ERK1/2 protein phosphorylation.	(Zhang et al. 2012)
Guanxin Kang	*Astragalus membranaceus* (Fish.) Bge*, Trichosanthes kirilowii* Maxim.*,Allium macrostemon* Bge.*, Leonurus japonicus* Houtt., *Salvia miltiorrhiza* Bge.*,Pinellia ternata* (Thunb.) Breit.	N/A	Play a protective role by regulating the apoptosis genes of cardiomyocytes.	(Qiu et al. 2012)
Gold Theragran Salvia Miltiorrhiza Prescription	*Polygonum multiflorum* Thunb., *Salvia miltiorrhiza* Bge., *Panax notoginseng* (Burk.) F. H. Chen	N/A	Reduce TNF-α, IL-1β mediated myocardial inflammatory response, promote the expression of myocardial PKC, inhibit the expression of iNOS in serum.	(Wang et al. 2007)
Hongqi formular	*Carthamus tinctorius* L., *Astragalus membranaceus* (Fish.) Bge	Safflor yellow, total saponins of astragalus	Reduce calcium overload, antioxidation, reduce inflammation.	(Wang Q and Shi 2017)
Huang Qi Tong Bi Decoction	*Astragalus membranaceus* (Fish.) Bge*, Angelica sinensis, Paeonia lactiflora* Pall.*, Ligusticum chuanxiong* Hort.*, Rehmannia glutinosa* Libosch.	N/A	Inhibit inflammation through the HMGB1/TLR/NF-κB pathway.	(Liu et al. 2019)
Huoxue Huatan Decoction	*Salvia miltiorrhiza* Bge.*, Astragalus membranaceus* (Fish.) Bge*, Panax notoginseng* (Burk.) F. H. Chen*, Ginkgo biloba* L.*, Hypericum monogynum* L.	Tanshinone IIA, Salvia Miltiorrhiza Polyphenols, Astragaloside IV, Panax Notoginseng Saponins	Reduce blood lipids, enhance PGC-1α-PPARα pathway activity, and then increase fatty acid β-oxidation to protect the structure and function of mitochondria.	(Lin et al. 2020)
Jiawei Danshen Decoction	*Salvia miltiorrhiza* Bge.*, Santalum album* L.*, Paeonia veitchii* Lynch, *Ligusticum chuanxiong* Hort.	N/A	Promote PKC activity, enhance NF-κB expression, decrease TNF-α, IL-2 and TXB2 levels, and inhibit inflammatory response.	(Huang et al. 2007)
Qidan Tongmai Tableton	*Astragalus membranaceus* (Fisch.) Bge*., Salvia miltiorrhiza* Bge., *Angelica sinensis* (Oliv) Diels.	Astragaloside, Salvia Miltiorrhiza Polyphenols, Safflor yellow-A, Carthamin	Regulate the expression of Bcl-2 and Bax and inhibit apoptosis and NF-κB protein expression in MI/R injury rat.	(Wang et al. 2007)
QiShenYiQi Pill	*Astragalus membranaceus* (Fisch.) Bge.*, Salvia miltiorrhiza* Bge.*, Panax notoginseng (*Burk.) F.H.Chen, *Dalbergia odorifera* T.Chen	Astragaloside IV, Salvianolic acid B, Notoginsenoside R1, Butein.	Upregulating PPARα/PGC-1α and fatty acid oxidation, reducing myocardial FFA and increasing ATP level.	(Tang et al. 2013)
Shuangshen Tongguan Recipe	*Panax ginseng* C. A. Mey*., Salvia miltiorrhiza* Bge.*, Corydalis yanhusuo* W. T. Wang	Total ginsenosides, total salvianolic acid, total alkaloids of rhizoma solanum	Inhibition of NF-κB signalling pathway, down-regulation of serum TNF-α and ICAM-1, and inhibition of Ca^2+^ overload of cardiomyocytes.	(Liu et al. 2005)
Tianlong Tongxin Tablet	*Rhodiola rosea, Rubiayunnanensis* (Franch.) Diels*,Salvia miltiorrhiza* Bge.*,Ligusticum chuanxiong* Hort*, Dracaena cochinchinensis* (Lour.) S. C. Chen	Salidroside	Inhibit platelet aggregation, reduce blood viscosity, and inhibit thrombosis.	(Li et al. 2019)
Wenyang Tongmai Decoction	*Panax notoginseng* (Burk.) F. H. Chen*, Panax ginseng* C. A. Mey.*, Citrus aurantium* L.*, Allium macrostemon* Bge.*, Cinnamomum cassia* Presl	N/A	Decrease the content of MDA, CK-MB and LDH, increase the level of SOD.	(Ma et al. 2008)
Yixinkang Capsule	*Salvia miltiorrhiza* Bge.*, Ligusticum chuanxiong* Hort*,Astragalus membranaceus* (Fish.) Bge	N/A	Improve SOD activity after reperfusion and enhance the ability to scavenge oxygen free radicals.	(Han et al. 2001)
Yixinyin	*Astragalus membranaceus* (Fish.) Bge*, Cinnamomum cassia* Presl*, Salvia miltiorrhiza* Bge.,	N/A	Reduce myocardial energy consumption, remove oxygen free radicals, and reduce the accumulation of calcium ions in cells.	(Wang et al. 2005)

### Chinese herbs considered to inhibit MI/R injury through anti-inflammatory effects

Inflammation plays a key role in MI/R injury. Previous studies (Wang et al. 2006) have found that the levels of inflammatory cytokines are directly related to the amount of damage to heart function and the number of necrotic cells after ischaemia. Among the 71 species of Chinese herbs accepted for this review, 27 species were found to inhibit inflammation in MI/R injury (Table 1). Among them, Compositae plants accounted for the highest proportion (22.2%), followed by Leguminosae plants (11.1%).

Reductions in cytokine levels are major anti-inflammatory effects of TCMs in the treatment of MI/R injury (Nos. 2 and 3 in Figure 1). As shown in Table 1, 13 species of Chinese herbs exert cardioprotective effects by regulating the levels of intracellular cytokines, including *Rosa rugosa* Thunb. (Rosaceae) (Hou et al. 2016), *Dioscorea zingiberensis* C. H. Wright (Dioscoreaceae) (Ebrahimi et al. 2014) and others. Among them, plumbagin extracted from *Plumbago zeylanica* L. (Plumbaginaceae) was used to prevent heart diseases in ancient times (Luo et al. 2010; Sheeja et al. 2010). Plumbagin induces activation of Nrf2 and reduces the expression of cytokines (MCP-1, IL-6, IL-8, and TNF-α) to return inflammation markers to normal levels (Wang et al. 2016). Valerian extract from *Valeriana officinalis* L. (Valerianaceae) (Xue et al. 1988) causes central sedation, exerts anti-arrhythmia effects, increases coronary blood flow, and reduces the scope of MI. Valerian extract can inhibit TNF-α production by monocytes/macrophages, reducing the expression of neutrophil (polymorphonuclear neutrophil, PMN) adhesion molecules, and thereby reducing the accumulation of PMNs in ischaemic regions in rabbits (Yin et al. 2000).

Some TCMs exert important therapeutic effects on MI/R injury by inhibiting the NF-κB signalling pathway (No. 4 in Figure 1). The increased production of ROS and pro-inflammatory cytokines resulting from myocardial ischaemia and hypoxia activates NF-κB (Karin and Greten 2005), and continuous activation of NF-κB leads to the expression of inflammatory cytokines, finally leading to cell death (Hamid et al. 2011). Caffeoylquinic Acid Derivatives Extract (AE) from *Erigeron multiradiatus* (Lindl.) Benth. (Compositae) significantly inhibits MI/R-induced injury by decreasing myocardial infarct size, reducing CK and LDH activity, and preventing ST-segment depression in a dose-dependent manner *in vivo*, by suppressing the myocardial inflammatory response and blocking the NF-κB and JNK activation pathways (Zhang et al. 2016). Gypenoside (GP), the prominent compound in *Gynostemma pentaphyllum* (Thunb.) Makino (Cucurbitaceae), can effectively increase the viability of damaged myocardial cells and decrease NF-κB relative binding activity. GP blocks NF-κB p65 translocation into the nucleus and inhibits downstream pro-inflammatory, showing enormous promise as a treatment agent for reperfusion injury (Yu et al. 2016). Additionally, *Bacopa monnieri* (Linn.) Wettst. (Scrophulariaceae) (Srimachai et al. 2017), *Sinomenium acutum* (Thunb.) Rehd. et Wils. (Menispermaceae) (Xu F 2018) and *Astragalus membranaceus* (Fisch) Bunge (Leguminosae) (Lu et al. 2015) act on the NF-κB signalling pathway to inhibit inflammation.

Some pro-inflammatory genes and adhesion molecule-encoding genes such as *ICAM-1*, are downstream of the NF-κB pathway, and an inflammatory response occurs when they are triggered (downstream in No. 4 in Figure 1). Total flavone of *Abelmoschus manihot* L. (Malvaceae) (TFA) contains a total of 12 flavonoids, of which the chemical structures of 8 flavonoids have been identified (Li et al. 2001; Fan et al. 2003). TFA can downregulate the high expression of *ICAM-1* and inhibit myocardial inflammation *in vivo* (Fan et al. 2006). The protective effect of TFA against MI/R injury is similar to that of ischaemic preconditioning (IPC), an endogenous protective mechanism in the body that is difficult to implement in the clinic. The therapeutic effect of TFA is better than that of the clinical medication verapamil (0.8 mg/kg), which suggests that TFA preconditioning is a promising strategy to effectively reduce the myocardial damage caused by MI/R (Fan et al. 2006).

Furthermore, inhibition of the NLRP3 inflammasome is an effective treatment means for MI/R injury. Reperfusion injury can trigger activation of the NLRP3 inflammasome; accelerate the secretion of IL-1β, IL-18 and caspase-1; and then induce an inflammatory response and pyroptosis (Bian et al. 2020). Hydroxysafflor yellow A (HSYA) of *Carthamus tinctorius* L. (Compositae) can inhibit the NLRP3 inflammasome by regulating the AMPK/mTOR signalling pathway (No.1 in Figure 1) *in vitro* (Ye et al. 2020) and *in vivo* (Ye et al. 2020). The therapeutic effect of HSYA on MI/R injury is comparable to that of diltiazem hydrochloride tablets (DTZ), a positive control. Studies have shown that HSYA may be a promising drug to prevent MI/R injury; therefore, its pharmacokinetics and toxicity should be further explored in the future. *Erigeron breviscapus* (Vant.) Hand.-Mazz. (Compositae) (Xu et al. 2020) and *Artemisia annua* L. (Compositae) (Wang et al. 2020) also exert myocardial protective effects by inhibiting the NLRP3 inflammasome.

### Chinese herbs considered to inhibit MI/R injury by reducing oxidative stress

We have summarised 35 species of Chinese herbs that reduce the oxidative stress state of cardiomyocytes to exert myocardial protection in Table 2. The most represented families among these species are the Leguminosae family (14.3%), the Compositae family (11.4%), the Polygonaceae family (8.6%) and the Rutaceae family (5.7%).

During oxidative stress in myocardial cells, neutrophil inflammatory infiltration and protease secretion increase, and a large number of oxidative intermediates are produced (Yellon and Hausenloy 2007). Most antioxidant effects of herbs involve scavenging of free radicals and enhancement of the activity of antioxidant enzymes (Nos. 10 and 11 in Figure 1). *Panax notoginseng* (Burk.) F. H. Chen (Apiaceae) is commonly used in China and was used as early as the publication of the *Compendium of Materia Medica*, as its use is recorded by Shizhen Li (Li et al. 2009). Notoginsenoside R1 (NGR1) is a new saponin extracted from *Panax notoginseng* that can reduce ROS levels in MI/R injury, participate in antioxidative stress mechanisms and restrain endoplasmic reticulum stress (ERS) (No.18 in Figure 1). Latifolin, a new flavone extracted from *Dalbergia odorifera* T. Chen. (Leguminosae), has the functions of removing blood stasis, regulating qi and relieving pain (Li et al. 2017; Zhang et al. 2019). An *in vitro* study has shown that latifolin significantly reduces the ROS content in H9c2 cells after hypoxia and reoxygenation (H/R), the mechanism of which may be related to activation of the Nrf2/HO-1 pathway (No. 9 in Figure 1). *Cassia mimosoides* L. (Leguminosae), is used as a food and tea (Yamamoto et al. 2000). In TCM, the whole plant is often used. An *in vivo* study (Lim and Lee 2012) has revealed that administration of the methanol extract of *Cassia mimosoides* reduces the infarct size in reperfusion injury-induced size of myocardial infarction MI by up to 28.3%. The methanol extract of *Cassia mimosoides* prevents MI/R injury mainly by scavenging ROS (No. 10 in Figure 1) and then blocking the apoptotic cascade. It is hoped that a new drug to prevent MI/R injury will be developed as a consequence of the identification of the active components of *Cassia mimosoides*. extract. Rutin, a natural flavonoid glycoside, is the main active ingredient of *Sophora japonica* L. (Leguminosae). In an antioxidant activity assay with 1, 1-diphenyl-2-picrylhydrazine (DPPH), 100 μM rutin was found to scavenge 82.62% ± 0.91 of radicals. *In vivo* and *in vitro* studies have shown that rutin can treat MI/R injury mainly by altering hemodynamic factors and enhancing the activity of the antioxidant enzymes superoxide dismutase (SOD) and DPPH (No. 11 in Figure 1) (Bhandary et al. 2012). A comparison of the therapeutic effects of five isoflavones on MI/R injury, including biochanin A, daidzein, genistein, quercetin and rutin, has suggested that rutin is the most protective isoflavone (Bhandary et al. 2012). In addition, herbs such as *Arctium lappa* L. (Compositae) (Chen et al. 2020), *Silybum marianum* (Linn.) Gaertn. (Compositae) (Cao et al. 2017), *Magnolia officinalis* Rehd.et Wils. (Magnoliaceae) (Tan et al. 2019), *Schisandra chinensis* (Turcz.) Ball (Magnoliaceae) (Zhang et al. 2017) can reduce ROS levels and increase antioxidants levels (Table 2).

### Chinese herbs considered to inhibit MI/R injury by reducing Ca^2+^ overload

Application of drug antagonists targeting Ca^2+^ channels, mitochondrial Ca^2+^ single transmitters and Na^+^-H^+^ exchange carriers to reduce the intracellular Ca^2+^ concentration can effectively reduce the area of MI by more than 50% (Ussher and Lopaschuk 2009). However, there have been few relevant studies in this area, and only *Mollugo pentaphylla* L. (Aizoaceae) (Li et al. 2016) has been found to reduce intracellular calcium overload, as shown in No. 12 in Figure 1 and Table 3. The whole plant of the grass *Mollugo pentaphylla* is used as a medicine with heat-clearing and detoxifying effects. Modern medical research has shown that *Mollugo pentaphylla* has anticancer, anti-arrhythmia, antihypertensive and other pharmacological effects (Liu KY 2009). Extract of *Mollugo pentaphylla* can effectively reduce the incidence of arrhythmia in rats, improve the activity of Ca^2+^-ATPase in myocardial tissue and maintain the levels at magnitudes comparable to those in sham-operated rats(Li et al. 2016), suggesting that *Mollugo pentaphylla* extract has a certain protective effect against Ca^2+^ overload in MI/R injury.

### Chinese herbs considered to inhibit MI/R injury by regulating mitochondrial energy metabolism

Reperfusion leads to intracellular Ca^2+^ overload, causing mitochondrial Ca^2+^ overload, which in turn prompts the opening of the MPTP and causes mitochondrial dysfunction (Hausenloy and Yellon 2013). We found eight kinds of Chinese herbs that reduce MI/R injury by regulating mitochondrial energy metabolism (Table 3). When reactive aldehydes are produced by oxidative stress due to sudden reperfusion of the damaged myocardium, aldehyde dehydrogenases (ALDHs) in mitochondria can convert the reactive aldehydes into harmless acids. Piperlonguminine (PPLG) is a kind of natural alkaloid extracted from *Piper longum* L. (Piperaceae). In a study in which a MI/R injury rat model was constructed and rat myocardial mitochondria were isolated (Yoval-Sánchez et al. 2020), PPLG was proved to reduce the levels of lipid peroxidation products, activate ALDHs to maintain mitochondrial function (No. 15 in Figure 1), and protect cardiomyocytes and tissues during MI/R injury.

Activation of mitoKATP channels can regulate Ca^2+^ uptake disorder and prevent MPTP opening and ROS formation to resist myocardial ischaemia, which is an important way to improve myocardial injury with drugs (Testai et al. 2021). *National herbal medicine assembly* records the use of *Dioscorea zingiberensis* for ‘the treatment of early boils ulcer, bee sting, appendicitis.’ Diosgenin is extracted from the rhizomes of *Dioscorea zingiberensis*, and an *ex-vivo* MI/R injury model study found that preconditioning with diosgenin (0.001 μM) can not only reduce the production of inflammatory mediators but also possibly provide myocardial protection by activating mitoKATP channels (No. 13 in Figure 1) (Ebrahimi et al. 2014). Notably, excessive *Dioscorea zingiberensis* may cause dizziness and other toxic phenomena, so it is critical to study the toxicology of diosgenin before developing a new drug. In addition, naringin (Nari), a flavonoid obtained from the dry outer layer of *Citrus maxima* (Burm.) Merr. (Rutaceae), has been documented to dose-dependently activate KATPs, especially mitoKATP, to exert a protective effect on damaged myocardia (Meng et al. 2016).

The ATP content and mitochondrial membrane potential can reflect the level of cardiomyocyte energy metabolism (He et al. 2021). Some medicinal plants protect mitochondria directly by promoting mitochondrial ATP synthesis, improving mitochondrial membrane potential and increasing Na^+^-K^+^-ATPase and Ca^2+^-Mg^2+^-ATPase activity (Takahashi et al. 2021). In particular, apigenin-8-C-β-d-glucopyranoside (vitexin), a bioactive flavonoid compound, can be isolated from the dried leaves of *Crataegus pinnatifida* Bge. (Rosaceae) and has been well studied. *In vivo* and *in vitro* (Xue et al. 2020), transmission electron microscopy imaging and mitochondrial isolation have shown that vitexin can reduce ROS levels, increase ATP content (No. 14 in Figure 1) and promote the elevations in matrix metalloproteinase (MMP) levels in MI/R injury. Vitexin can alleviate mitochondrial damage, maintain the dynamic balance of mitochondria, reduce the area of MI, and improve the function of the damaged myocardium; thus, it has therapeutic potential for MI/R injury.

### Chinese herbs considered to inhibit MI/R injury by reducing apoptosis, autophagy and necrosis

We found 19 species of Chinese herbs that have a regulatory effect on apoptosis (Table 4). Caspase-3, Bax, Bcl-2 and other apoptosis-related proteins can directly reflect apoptosis. Many herbs regulate these apoptosis-related proteins to inhibit apoptosis (No. 16 in Figure 1), such as *Paeonia veitchii* Lynch (Ranunculaceae) (Wang K and Hu 2020), *Pueraria lobata* (Willd.) Ohwi (Leguminosae) (Fan HX and Zhang et al. 2017), and *Curcuma longa* L. (Zingiberaceae) (Mohanty et al. 2006). The mitogen-activated protein kinase (MAPK) signalling pathway is an important pathway causing myocardial cell injury and apoptosis (Li et al. 2016). Two medicinal plants [*Diospyros kaki* Thunb. (Ebenaceae) (Meng et al. 2019), *Gastrodia elata* Bl. (Orchidaceae) (Zhang et al. 2019)] can reduce the rate of myocardial apoptosis and the size of MI by regulating the MAPK/ERK1/2 signalling pathway (No. 6 in Figure 1). Furthermore, ERS is a relatively newly identified mechanism of apoptosis regulation (No. 18 in Figure 1). The endoplasmic reticulum plays irreplaceable roles in protein folding, transport and secretion (Xu et al. 2005). ERS is triggered by intracellular homeostasis imbalance, and continuous ERS induces cells to enter the apoptotic program, which hampers cell function (Fernández et al. 2015). The main active ingredient of *Dracaena cochinchinensis* (Lour.) S. C. Chen (Liliaceae) is resina draconis (RD), which is extracted from the dried trunk of the plant. Studies have shown that RD can serve as an antioxidant and preservative compound (Choy et al. 2008). In a MI/R injury model (Yang et al. 2019), RD (1 mg/mL) has been found to effectively increase SOD activity and reduce MDA content almost to normal levels and significantly reduce the levels of GPR78, CHOP and other apoptosis-related proteins. These findings suggest that RD inhibits ER-induced apoptosis in MI/R injury by regulating miR-423-3p and its target ERK. For protecting the myocardium from reperfusion injury by targeting ERS, *Allium fistulosum* L. (Liliaceae) (Li 2019), *Schisandra chinensis* (Zhang et al. 2017) and *Paeonia lactiflora* (Zheng YP and Liu et al. 2019) are also available.

The process of autophagy has been conserved throughout cell evolution (Fujiwara et al. 2013); however, excessive autophagy caused by reperfusion exacerbates tissue damage (Ma et al. 2015). We screened three herbs [*Coptis chinensis* Franch. (Ranunculaceae) (Zhu et al. 2020), *Magnolia officinalis* (Tan et al. 2019) and *Gardenia jasminoides* Ellis (Rubiaceae) (Luo et al. 2020)] that regulate autophagy in MI/R injury (Table 5 and No. 19 in Figure 1). Berberine (BBR), the main active ingredient of *Coptis chinensis*, can effectively reduce the autophagic flux of H9c2 cells subjected to H/R and improve mitochondrial function (Zhu et al. 2020). Moreover, *in vivo* and *in vitro* studies have shown that geniposide, an organic compound extracted from the dried and ripe fruits of *Gardenia jasminoides* is, can effectively reduce the MI size and H/R-induced autophagosome formation. The possible mechanism may involve inhibition of autophagy through the AKT/mTOR signalling pathway (Luo et al. 2020).

During reperfusion, TNF-α and other inflammatory factors bind to their receptors to activate programmed necrosis. Eventually, necrosomes composed of RIP1, RIP3 and MLKL are formed to execute cell necrosis (Pasparakis and Vandenabeele 2015). We found that three species of Chinese herbs can regulate necrosis caused by MI/R injury (Table 5 and No. 20 in Figure 1), including *Scutellaria baicalensis* Ceorgi (Labiatae) (Wang et al. 2020), *Bauhinia championii* Benth. (Leguminosae) (Zhang et al. 2016) and *Arctium lappa*. (Chen et al. 2020). In particular, arctiin, an extract of *Arctium lappa*, not only plays an antioxidant role in the treatment of MI/R injury but also has an inhibitory effect on necrosis in H9c2 cell subjected to H/R (Chen et al. 2020). Detection of the protein levels of RIPK1/pRIPK1, RIPK3/pRIPK3 and MLKL/pMLKL, has confirmed that arctiin inhibits cell necrosis by inhibiting the production of necrosis-related proteins. According to bioinformatics data, arctiin may also directly target RIPK1 and/or MLKL to prevent necrosis in MI/R injury.

### Potential Chinese medicines considered to protect against MI/R injury

Several Chinese plants have potential protective effects against MI/R injury (Table 6), including *Camellia oleifera* Abel (Theaceae) (Lai et al. 2004) and *Rosmarinus officinalis* L. (Labiatae) (Zhang et al. 2017). Sasanquasaponin (SQS) is an effective extract of *Camellia oleifera* whose main structure is similar to that of some ginseng saponins (Liu CX and Xiao 1992; Attele et al. 1999). *In vitro* studies (Lai et al. 2004) have shown that SQS can inhibit arrhythmia during MI/R and may play a protective role in the myocardium by regulating intracellular Cl^-^ homeostasis. *Rosmarinus officinalis* is a traditional herb with abundant application value. Rosemary extract (MDX60) is derived from *Rosmarinus officinalis*; the main component is carnosic acid (60%). A study on MI/R model rats (Zhang et al. 2017) has shown that MDX60 can reduce the MI size. These potential medicinal plants have not yet been used in clinical practice, and more *in vivo* and *in vitro* studies are needed to support their use. Further exploration of their specific mechanisms may lead to new measures for the treatment and prevention of MI/R injury.

### TCM compound prescriptions considered to have therapeutic effects on MI/R injury

The compatibility of TCMs with other therapeutics can often expand the range of treatments and enhance the efficacy of drugs, which may help maximise the impact on MI/R injury given its complicated mechanisms. Huoxue Huatan Decoction has been used for the treatment of cardiovascular diseases for over 20 years. It is composed of *Salvia miltiorrhiza*, *Astragalus membranaceus*, *Panax notoginseng, Ginkgo biloba* L., *Trichosanthes kirilowii* Maxim. (Cucurbitaceae), *Allium macrostemon* Bunge (Liliaceae). and *Ziziphus jujuba* Mill. (Rhamnaceae). *In vivo* studies (Lin et al. 2020) have shown that Huoxue Huatan Decoction can regulate lipid metabolism in MI/R damaged hyperlipidaemia rats in a dose-dependent manner, improve mitochondrial energy disorder through the PGC-1α-PPARα signalling pathway, promote the expression of PGC-1α-NRF1-mtTFA and increase T-SOD levels to protect the heart. Compound Danshen tablet promotes blood circulation and removes stasis, regulating qi and relieving pain (Fan et al. 2017; Liang et al. 2019; Zheng CM 2019). It is mainly used for the clinical treatment of cardiovascular and cerebrovascular diseases and has definitive curative effects (Liang et al. 2019). It has even been used as a positive control in efficacy studies on other TCMs (Fan HX and Zhang et al. 2017). The major components include *Salvia miltiorrhiza*, *Panax notoginseng,* and *Dryobalanops aromatica* Gaertn. f. (Dipterocarpaceae). Among them, *Salvia miltiorrhiza* plays a major role; this drug is also known as the "monarch drug" in TCM. An *in vivo* study (Li et al. 2020) has shown that compound Danshen tablet can significantly reduce ST segment elevation; the myocardial ischaemia rate; and the levels of CK, LDH, AST and other myocardial enzymes in serum while increasing the levels of NO and eNOS (a kind of cardiovascular protective molecule) in MI/R injury model rats. Additionally, the therapeutic effect of compound Danshen tablet is better than that of the single medicines in improving myocardial ischaemia (Li et al. 2020). More information on Chinese compound prescriptions is listed in Table 7. All of the prescriptions have been proven to have therapeutic efficacy against MI/R injury. Among these prescriptions, *Salvia miltiorrhiza* and *Astragalus membranaceus* are the most commonly used monarch drug. Tanshinone IIA in *Salvia miltiorrhiza* and astragaloside IV in *Astragalus membranaceus*, which have anti-inflammatory and antioxidative effects (Li et al. 2017; Ren et al. 2019; Yuan et al. 2020), are promising agents for MI/R injury.

## Conclusions and outlook

This article reviews advances in research on the use of TCMs in the treatment of MI/R injury from 1980 to 2020. We collected and summarised information on the families, Latin binomials, parts used, active compounds, doses, experimental animal models, pharmacological mechanisms and references in tables to provide theoretical support for screening and developing safe, efficient and low-toxicity drugs for MI/R injury. Among the 71 species (40 families) identified as Chinese herbs used to treat MI/R, Compositae herbs accounted for the largest proportion at 11.3%. The second most popular family was Leguminosae, accounting for 10% of species. Compositae plants mostly inhibit MI/R injury by exerting anti-inflammatory effects, while Leguminosae plants primarily exert myocardial protective effects by reducing oxidative stress. Among the TCMs whose active ingredients have been identified, we found that the myocardial protective effect is mainly attributed to flavonoids, such as baicalein in *Scutellaria baicalensis* (Wang et al. 2020), rutin in *Sophora japonica* (Bhandary et al. 2012) and silybin in *Silybum marianum* (Cao et al. 2017); anthraquinones are the second most important components for myocardial protection. Flavonoids can scavenge ROS such as superoxide and hydrogen peroxide free radicals to reduce oxidative stress; inhibit the pro-inflammatory cytokines production and restrict inflammatory mediator levels, enhance Na^+^/K^+^-ATPase activity and reduce intracellular Ca^2+^ overload. These compounds, which have C6-C3-C6 structures, can exert effective vasodilatory, antioxidant, anti-inflammatory, and antiapoptotic effects in the damaged myocardium. Structural modification of flavonoids and anthraquinones is expected to lead to the development of new effective therapeutic drugs for MI/R injury. In addition, two species with potential therapeutic effects and 18 types of TCM compound prescriptions were included in the current review.

TCMs have the advantages of low toxicity, few side effects, stable curative effects, and the ability to act on multiple pathways. In addition to crude extracts and identified effective monomers, Chinese compound prescriptions have shown significant therapeutic potential in *in vivo* and/or *in vitro* basic research, suggesting that TCMs have potential treatment value for MI/R injury. TCMs are widely used globally, but the complexity of the chemical compositions of TCM compound formulas makes replication and pharmacological research difficult. Tremendous efforts need to be made to address this issue. More in-depth studies on the active ingredients, pharmacokinetics and drug toxicity of TCMs can be conducted, and more effective drugs with fewer side effects can be screened. Additionally, structural modification of the main active monomers is a promising strategy to develop new efficient drugs for MI/R injury. Importantly, to demonstrate the clinical activity of TCMs in MI/R injury, large randomised trials are required.
